# In Vitro and In Vivo Characterization of Ultraviolet Light C-Irradiated Human Platelets in a 2 Event Mouse Model of Transfusion

**DOI:** 10.1371/journal.pone.0079869

**Published:** 2013-11-01

**Authors:** Li Zhi, Xuan Chi, Jaroslav G. Vostal

**Affiliations:** Laboratory of Cellular Hematology, Division of Hematology, OBRR, Center for Biologics Evaluation and Research, Food and Drug Administration, Bethesda, Maryland, United States of America; Royal College of Surgeons, Ireland

## Abstract

UV-based pathogen reduction technologies have been developed in recent years to inactivate pathogens and contaminating leukocytes in platelet transfusion products in order to prevent transfusion-transmitted infections and alloimmunization. UVC-based technology differs from UVA or UVB-based technologies in that it uses a specific wavelength at 254 nm without the addition of any photosensitizers. Previously, it was reported that UVC irradiation induces platelet aggregation and activation. To understand if UVC-induced changes of platelet quality correlate with potential adverse events when these platelets are transfused into animals, we used a 2-event SCID mouse model in which the predisposing event was LPS treatment and the second event was infusion of UVC-irradiated platelets. We analyzed lung platelet accumulation, protein content in bronchoalveolar lavage fluid as an indication of lung injury, and macrophage inflammatory protein-2 (MIP-2) release in mice received UVC-irradiated or untreated control platelets. Our results showed UVC-irradiated platelets accumulated in lungs of the mice in a dose-dependent manner. High-doses of UVC-irradiated platelets were sequestered in the lungs to a similar level as we previously reported for UVB-irradiated platelets. Unlike UVB-platelets, UVC-platelets did not lead to lung injury or induce MIP-2 release. This could potentially be explained by our observation that although UVC treatment activated platelet surface αIIbβ3, it failed to activate platelet cells. It also suggests lung platelet accumulation and subsequent lung damage are due to different and separate mechanisms which require further investigation.

## Introduction

Transfusion-transmitted infections have been significantly reduced in recent years due to improved donor screening and testing for blood borne pathogens. However, pathogens including bacteria, low-titer viruses, parasites, and novel emerging pathogens have the capacity to escape detection from conventional blood bank screening and present an infectious risk to transfusion recipients[1-5]. To address this issue, pathogen reduction technology (PRT) has been developed and applied to blood products before storage, with the aim of rendering residual or undetected pathogens in the unit noninfectious. 

Currently three UV technologies, all targeting nucleic acids for bacterial, viral, protozoal and leucocyte inactivation in platelet concentrates, have been developed and are in clinical use or have reached clinical trial evaluations in Europe. These methods include the Cerus INTERCEPT system, which uses UVA (320-400 nm) activation of a psoralen derivative (Amotosalen) to cross-link the nucleic acids of pathogens and prevent their replication [6], the Terumo BCT MIRASOL system which uses broad band UV (UVA+UVB, 280-400 nm) to activate Riboflavin (vitamin B2), which associates with nucleic acids and mediates an oxygen-independent electron transfer process, leading to the modification of nucleic acids [7]. Both approaches rely on the use of a photosensitizer (Amotosalen or Riboflavin) to irreversibly damage DNA/RNA, which may raise the risk of photoproducts-associated adverse effects such as immune reactions, toxicity or even carcinogenicity [8,9]. Even after passing through phase III clinical trials, toxicity may not be revealed until large-scale patient population exposure is seen [10]. 

Recently, MacoPharma introduced THERAFLEX UV-Platelets system which is based solely on UVC of a specific wavelength (254 nm) without addition of any photosensitizers [11]. Short-wave UV light is directly absorbed by nucleic acids of pathogens and leucocytes and results in formation of cyclobutane pyrimidine and pyrimidine pyrimidone dimers, which block the elongation of nucleic acid transcripts [12]. UVC light at 254 nm coincides closely with the maximum absorption of DNA/RNA (260 nm) but is near the minimum absorption of proteins and theoretically it would cause minimal damage to plasma and platelet proteins [13]. Since UVC light is quenched in turbid or protein-containing solutions, the THERAFLEX UV-Platelets system is designed to overcome this obstacle by suspending platelets in 65% additive solution (SSP^+^), exposing platelets to UVC light over a large surface area (19 x 38 cm illumination bag), and agitating the platelets during exposure. 

The successful application of PRT to transfusion products relies on the balance between the efficacy of pathogen reduction and the ability to preserve blood cell quality. Current studies suggest all PRT procedures have a negative impact on the platelet storage lesion and appear to moderately increase the activation and metabolic activity of platelets [14,15]. Although the relationship between the in vitro platelet activation and in vivo function after transfusion remains controversial, current evidence indicates a consistent reduction of in vivo recovery and survival of PRT-treated platelets when stored for 5 days and compared with untreated platelets in healthy volunteers in platelet radiolabeling studies : INTERCEPT-platelets have 16% lower recovery and 20% lower survival [16]; MIRASOL-platelets have 25% lower recovery and 27% lower survival [17]; and THERAFLEX-platelets have 26% lower recovery and 29% lower survival [15]. 

In addition to loss of viability recent clinical trials also revealed potential respiratory adverse events associated with INTERCEPT and MIRASOL-treated platelets [18,19]. To further understand the effect of UV light on platelet quality and in vivo function after transfusion, we developed a two-event mouse model using severe combined immunodeficient (SCID) mice where the first event is LPS priming and the second event is transfusion of human platelets [20]. The absence of functional T and B cells in SCID mice allows delayed clearance of transfused human platelets from circulation, and provides an excellent tool to evaluate the recovery and survival, as well as potential adverse events associated with UV-treated platelets. In this study, we investigated the effects of UVC light on platelet in vitro and in vivo performance. We found that UVC irradiation causes significant platelet modification that leads to aggregation and is associated with reduction of in vivo recovery and enhanced lung platelet accumulation in the recipient animals. However, unlike with UVB-treated platelets [20,21], we did not observe induction of chemokine release or generation of acute lung injury in LPS-primed mice transfused with even high-dose UVC-irradiated platelets.

## Materials and Methods

### Ethics statement

Animal protocol (#2005-15) was approved by the Center for Biologics Evaluation and Research Intramural Animal Care and Use Committee at Food and Drug Administration (FDA). Blood products were collected at the NIH Division of Transfusion Medicine under full institutional review board approval (NIH Clinical Center IRB protocol #00- CC-0168). The FDA Research Involving Human Subjects Committee (RIHSC, John J. McCormick, MD, Chair) reviewed the study under protocol #03-084B and found it to be exempt from IRB and RIHSC review under 45CFR 46.101 (b) (4): "Research involving the collection or study of existing diagnostic specimens, where the subjects cannot be identified, directly or through identifiers linked to the subjects." 

### Materials

Monoclonal antibodies (mAbs) used for flow cytometry were purchased from BD Biosciences (San Jose, CA) and include fluorescein isothiocyanate (FITC) conjugated PAC-1 and anti-human CD41a (clone HIP8), phycoerythrin (PE) conjugated anti-human P selectin (CD62P, clone AK-4) and matched isotype control antibodies. mAbs and reagents used for immunofluorescence staining include mouse anti-human CD41a (clone H1P8, ABBIOTEC, San Diego, CA), Alexa Fluor 568-conjugated goat anti-mouse IgG1 and TO-PRO-3 (Invitrogen, Carlsbad, CA). Unless indicated otherwise, all other reagents were obtained from Sigma (St. Louis, MO).

### Mice

6- to 8- week-old female CB-17 severe combined immunodeficient (SCID) mice were obtained from the National Cancer Institute Frederick Animal Production Program and maintained in a pathogen-free facility prior to experiments according to guidelines of the Animal Research Advisory Committee of National Institutes of Health. Animal protocols were approved by the Center for Biologics Evaluation and Research Intramural Animal Care and Use Committee at Food and Drug Administration. 

### Preparation of human platelets

Human platelets in plasma were collected by apheresis in ACDA (MCS+, Haemonetics, Braintree, MA) at the NIH Division of Transfusion Medicine under full institutional review board approval. The platelet-rich-plasma (PRP) was kept at room temperature on a Helmer PF96 platelet agitator (Helmer, Noblesville, IN) for 1 or 2 days before use. Platelets were centrifuged at 1,000 x *g* for 10 minutes at room temperature in the presence of 140 nM PGE_1_ to pellet the cells. The supernatant was removed and further centrifuged at 2,500 x *g* for 15 minutes to prepare platelet-poor-plasma (PPP). The remaining platelet pellet was gently resuspended in 35% (v/v) PPP/65% (v/v) PAS III solution (Intersol, Fenwal, Lake Zurich, IL) and adjusted to 1 x 10^9^/mL. The platelets were allowed to rest for 30 minutes at room temperature before use. For mouse intravenous injections, platelets were concentrated by centrifugation at 1000 x *g* for 10 minutes at room temperature in the presence of 1μM PGE_1_ and subsequently resuspended to 1 x 10^10^/mL with 35% PPP/65% PAS III. The concentrated platelets were rested at room temperature for 30 minutes before 100 uL was injected into the mice, resulting in transfusion of a total of 1 x 10^9^ human platelets into each mouse. The number of human platelets was counted with a Cell-Dyn 3700 blood cell counter (Abbott, Abbott Park, IL).

### UVC irradiation

10 mL platelets suspended in 65% PAS III was added to a polypropylene container (8.5 x 10 cm), resulting in a suspension depth of 1.2 mm. The platelet container without cover were irradiated from above with two UV bench lamps placed in parallel emitting shortwave UV at 254 nm (XX-series, UVP, Upland, CA) at constant intensity (about 7 mW/cm^2^) for 30 seconds or 3 minutes at room temperature, resulting in a UVC dose of 0.2 and 1.2 J/cm^2^ , respectively. The dose of UVC light delivered was measured with a photo radiometer with a UVC sensor (Model UVX, UVP, Upland, CA). Control platelets were placed in identical containers on the bench at room temperature for the same amount of time.

### 2-event mouse model

Mice were primed with an intraperitoneal (i.p.) injection of lipopolysaccharide (LPS, 3 mg/kg) two hours prior to being challenged with an intravenous (i.v.) tail vein injection with 1 x 10^9^ human platelets or PBS. 

### Measurement of platelet aggregation

The UVC-induced platelet doublet or aggregation formation was directly determined by measuring the decrease in the number of single platelets in the Cell-Dyn 3700 blood cell counter. Adenosine diphosphate (ADP) induced platelet aggregation response was measured on a Bio/Data PAP8E aggregometer (Horsham, PA) as described previously [21].

### Flow cytometry

Human platelets were mixed with CD62P PE, CD41 FITC, PAC-1 FITC mAb or corresponding isotype control antibodies for platelet activation evaluation. For evaluating in vivo recovery of human platelets in mice, about 15 uL of mouse blood was removed at 5 and 20 minutes and at 2, 4, 6 and 24 hours after platelet transfusion with heparinized capillary tubes using standard mouse tail clipping technique. The samples were mixed immediately with anti-human CD41 FITC mAb. The samples were incubated for 20 min at room temperature in phosphate-buffered saline (PBS) containing 0.3 % (v/v) bovine serum albumin (BSA). Samples were diluted 10-fold with PBS/0.3% BSA before examined on a FACSCalibur flow cytometer equipped with CellQuestPro software (Becton Dickinson, San Jose, CA), gating platelets with forward- and side-scatter settings. Data were analyzed with FlowJo software (TreeStar, Ashland, OR). 

### Immunohistochemistry

Mice were euthanized two hours after platelet injection by CO_2_ exposure. Mouse lungs were inflated by intratracheal injection of 1 mL 1:1 mixture of OCT compound/PBS. One lobe of the lung was tied off at the bronchus, removed and snap-frozen in OCT in an ethanol dry-ice bath, and sectioned at 10 µm. Cryosections were stained with anti-human CD41 monoclonal antibody as described previously [20]. The specificity of this antibody for human platelets was confirmed and it does not cross react with mouse platelets [22]. TO-PRO-3 (1:1000) was used for nuclear staining. All sections were photographed using a Zeiss LSM710 confocal microscope with a 63x/NA1.4 Plan-Apochromat oil objective (Carl Zeiss Inc, Germany). Three serial lung sections from at least five mice of each treatment group were used for analysis. Pixel quantification of the fluorescence signals was obtained using Adobe Photoshop CS3 (Adobe Systems, San Jose, CA) as previously described [23]. 

### Histology

Mice were euthanized two hours after platelet injection by CO2 exposure. Mouse lungs were inflated by intratracheal injection of 1 mL cold 4% paraformaldehyde. One lobe of the lung was tied off at the bronchus and excised, and the entire lobe was submerged in 4% paraformaldehyde and fixed overnight at 4 °C. Lungs were washed once for 5 minutes in PBS, placed in 70% ethanol, and sent for paraffin embedding and sectioning (Histoserv, Rockville, MD). Lung sections (7 μm) were stained with hematoxylin and eosin (Protocol Hema 3, Fisher Scientific, Kalamazoo, MI) and photographed using an Olympus CK40 microscope with an NA0.30 objective.

### Total protein measurement in bronchoalveolar lavage fluid (BALF)

Mice were euthanized two hours after platelet injection by CO_2_ exposure. The pleural cavity was surgically opened, and the trachea was cannulated with an 18 gauge blunt needle and lungs were lavaged three times with 1 mL PBS. About 2.2-2.5 mL BALF was routinely collected. The BALF was centrifuged at 200 x *g* for 15 minutes at 4 °C. The supernatant was removed and saved at -20°C for protein measurement. The total protein concentration in the BALF was measured using QuantiPro BCA Assay Kit (Sigma, St. Louis, MO) according to manufacturer’s instructions. 

### MIP-2 measurement by ELISA

Mice were euthanized three hours after platelet injection by CO_2_ exposure. About 500 µL mouse blood was collected by cardiac puncture and centrifuged at 1,500 x *g* for 15 minutes at room temperature. The plasma fraction was collected and frozen at -80 °C. Plasma and BALF MIP-2 concentration was measured using the mouse CXCL2/MIP-2 ELISA kit (DuoSet, R&D systems, Minneapolis, MN) according to manufacturer’s instructions.

### Statistical analysis

Results are reported as Mean ± SE. Significance was determined with Student’s *t* test with *P* values of less than 0.05 set to be statistically significant (* *p* < 0.05, ** *p* < 0.01, *** *p* < 0.001).

## Results

### UVC exposure induces platelet aggregation in a dose dependent manner

**Figure 1:**
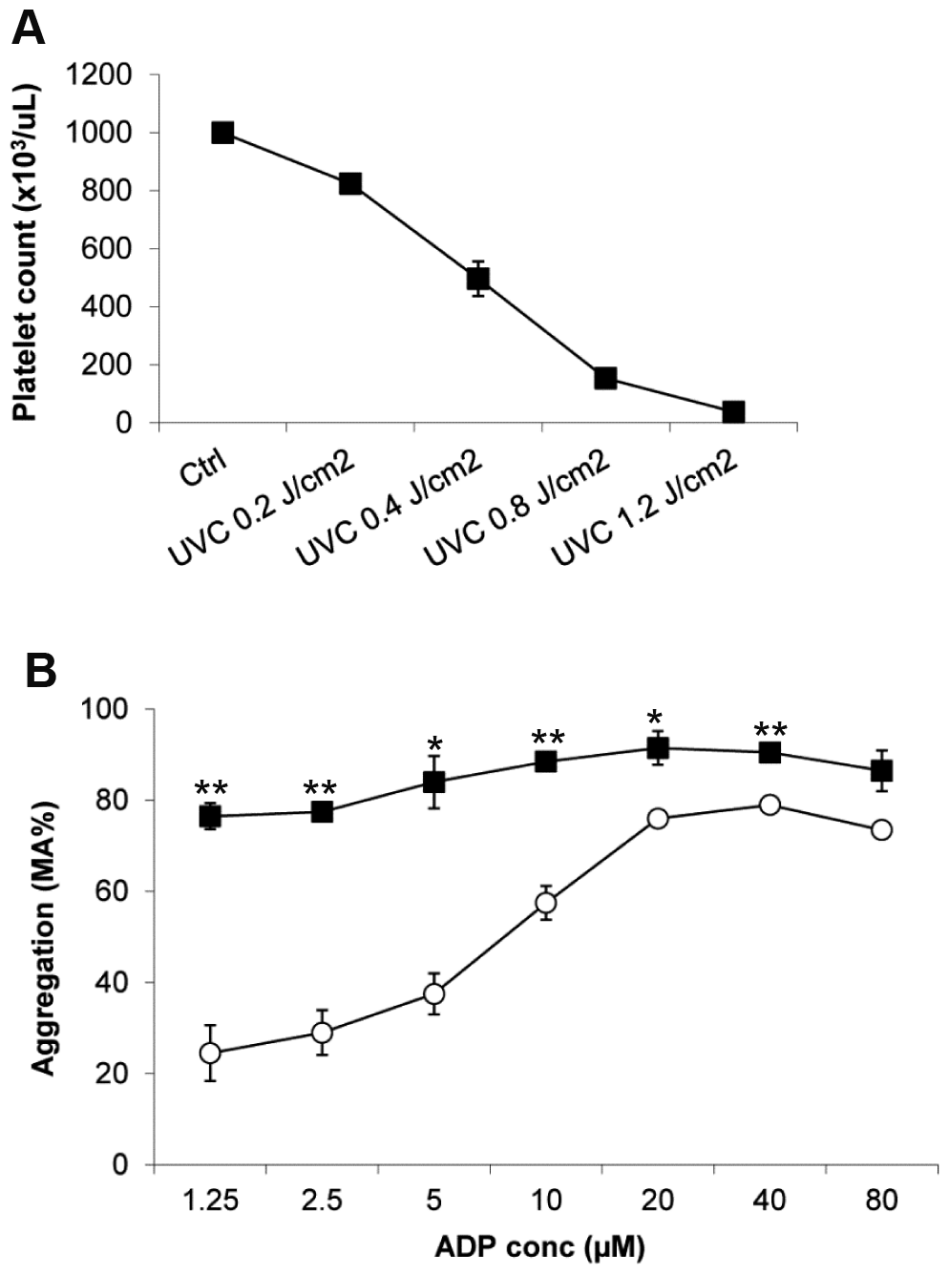
UVC irradiation induced platelet aggregation and potentiated ADP induced platelet aggregation. *A*) Human platelets (HPs) at 1 x 10^6^/µL were exposed to 0.2 (30 seconds), 0.4 (1 minutes), 0.8 (2 minutes), and 1.2 J/cm^2^ (3 minutes) UVC illumination. Single platelet count was measured immediately after light exposure on a Cell-Dyn 3700 blood cell counter. Mean ± SE, n=8. *B*) in vitro aggregation assay was performed with untreated (Ctrl, open circle) or UVC-exposed platelets (0.2 J/cm^2^, filled square). Y axis represents percent of maximal aggregation (%MA). Mean ± SE, n=3.

Previous studies suggested that UVC irradiation induced platelet aggregation [24]. When platelets were irradiated with a UVC dose of 0.15 J/cm^2^, platelet count decreased immediately with formation of large aggregates detectable under a light microscope [24]. To further quantitatively characterize the effect of low- and high-dose UVC irradiation on platelet aggregation, we exposed platelets at a concentration of 1 x 10^9^/mL suspended in 35% (v/v) plasma/65% PAS III solution to increasing doses of UVC light ranging between 0.2 and 1.2 J/cm^2^ (Figure 1A). Single platelet count was measured immediately after light exposure. We found that a UVC dose of 0.2 J/cm^2^ induced a slight decrease in single platelet count (14.6 %, 853.2 ± 10.7 vs 999.4 ± 11.9). When the UVC dose was increased to 0.4 J/cm^2^, a more significant decrease in single platelet count was observed (50.4 %, 496.2 ± 132.9 vs 999.4 ± 11.9). Further increasing the UVC dose to 0.8 and 1.2 J/cm^2^ led to formation of visible platelet aggregates in the platelet suspension, resulting in dramatic reduction of single platelet count (84.7 % and 96.3%, 153.1 ± 72 and 37.1 ± 19.8 vs 999.4 ± 11.9, respectively) as measured by the automatic cell counter. 

### UVC exposure increases platelet response to ADP

Our previous work showed that UVB light induced platelet aggregation and potentiated platelet responses to weak agonists such as ADP [21]. To test whether exposure to UVC light also increased the platelet response to ADP, we added increasing concentrations of ADP (1.25 to 80 μM) to untreated control and low-dose (0.2 J/cm^2^) UVC-irradiated platelets and characterized the platelet aggregation response with an aggregometer. To stimulate the biphasic aggregation in control platelets, 10 μM Epinephrine was added along with ADP to all control and UVC-platelet samples. As shown in Figure 1B, UVC-irradiated platelets showed significantly increased response to ADP stimulation, especially when low-concentrations of ADP were used. When stimulated with 1.25 μM ADP, UVC-platelets achieved 76.5 ± 5.0 % maximum aggregation (MA), whereas control platelets achieved 24.5 ± 10.6 % MA. UVC-platelets showed similarly increased sensitivity to ADP as a single agonist whereas control platelets exhibited a limited response (data not shown). 

### UVC irradiation activates platelet integrin αIIbβ3 without affecting P-selectin expression

**Figure 2:**
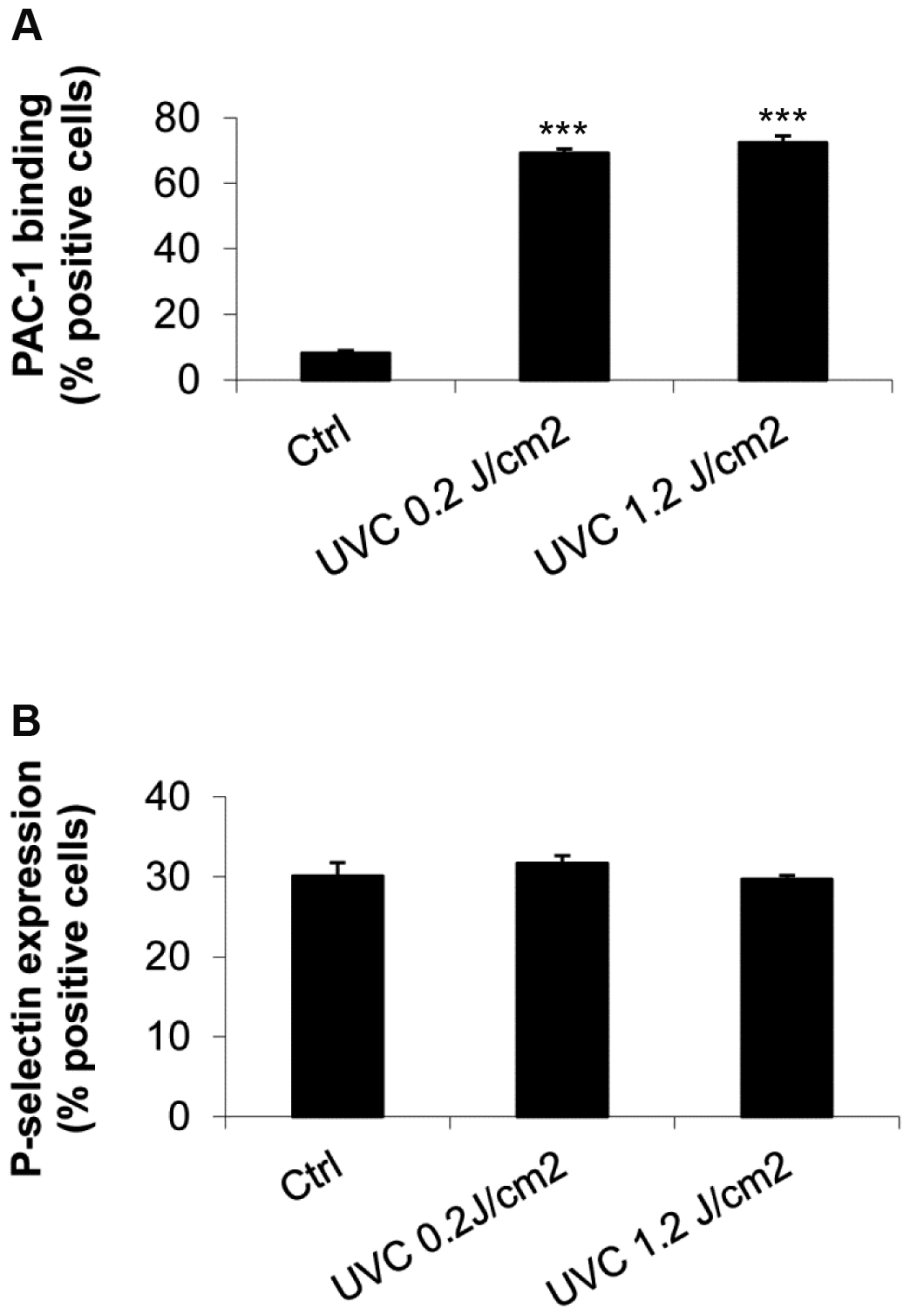
UVC irradiation of HPs activated αIIbβ3 integrin without affecting platelet surface P-selectin expression. Untreated and UVC irradiated HPs at varying UVC doses were labeled with PAC1-FITC or with a combination of CD41a-FITC and CD62P-PE and the binding was detected with a FACSCalibur cytometer. Shown here is flow cytometric quantification of the percentage of platelet cells positive for PAC-1 binding (*A*) or CD41a and CD62P staining (*B*). Mean ± SE, n=3-5.

Verhaar R et al. showed that increasing doses of UVC irradiation increased binding of PAC-1 monoclonal antibody to platelets [24]. This antibody selectively recognizes the high-affinity active conformation of platelet integrin αIIbβ3 [25-27]. We further characterized the effect of UVC irradiation on platelet activation by analyzing platelet PAC-1 binding and surface P-selectin expression. We found that, similar to UVB irradiation [21], UVC exposure of platelets did significantly increase PAC-1 binding (Figure 2A). At a dose of 0.2 J/cm^2^ UVC light, about 70% of platelets demonstrated PAC-1 binding (69.3 ± 2.2 % positive cells), whereas binding in untreated control platelets was barely detected (8.1 ± 1.9 % positive cells). When the UVC dose was increased by 6-fold to 1.2 J/cm^2^, no further increase in PAC-1 binding to UVC-platelets was observed under our experimental conditions. In contrast to the marked effect of UVC on activation of platelet αIIbβ3, we did not observe an effect of UVC on platelet surface P-selectin expression, which remained at about 30% of the cells positive after the platelets were exposed to 0.2 or 1.2 J/cm^2^ UVC irradiation (Figure 2B). 

### UVC-irradiated platelets show reduced in vivo recovery in SCID mice

**Figure 3:**
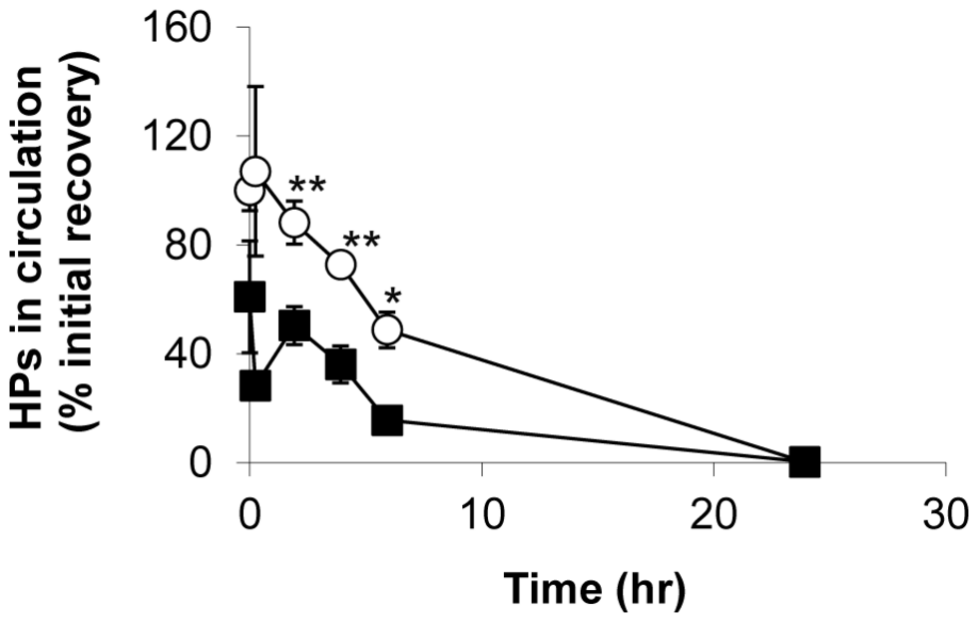
UVC-irradiated HPs showed reduced in vivo recovery in circulation of SCID mice. Approximately 1 x 10^9^ untreated (open circle) or UVC-irradiated HPs at a dose of 0.2 J/cm^2^ (filled square) were infused into SCID mice. Blood sampling was subsequently performed at indicated time points and the presence of human platelets in circulation positive for anti-human CD41a staining was detected by flow cytometry. Mean ± SE, n=5.

To assess the impact of UVC irradiation on platelets in vivo, we compared the survival and recovery of human platelets exposed to either a UVC dose of 0.2 J/cm^2^ or untreated control platelets in SCID mice circulation . Approximately 1 x 10^9^ human platelets were transfused into each animal. Using the percentage of cells positive for the specific human CD41 antibody staining in the platelet acquisition gate of control platelets at 5 minutes after platelet infusion as 100 % recovery, the recovery of UVC and control platelets was evaluated at 5 and 20 minutes and at 2, 4, 6, and 24 hours after platelet infusion (Figure 3). We found that the recovery of UVC platelets in circulation at 5 minutes after infusion was 39.1 % lower than that of control platelets (60.9 ± 20.5% vs 100 ± 7.4%). The presence of UVC-platelets in circulation was quickly reduced within 20 minutes, resulting in 78.6 % lower recovery than control platelets (28.4 ± 3.6% vs 107 ± 31.1%). By 2 hours after infusion, the level of UVC platelets in circulation increased and the difference in recovery between UVC and control platelets persisted at about 35 % at 2, 4 and 6 hours (50.5 ± 7% vs 88.2 ± 7.9%, 36.2 ± 6.8% vs 72.7 ± 3%, and 15.6 ± 0.3% vs 48.9 ± 6.6%, respectively). By 24 hours after infusion, the recovery of both UVC and control platelets approached zero. The approximate t_1/2_ of human platelets in mouse circulation estimated graphically and defined as the time to reach 50 % recovery, was about 2 hours for UVC platelets and 6 hours for control platelets in SCID mice. 

### Transfusion of UVC-irradiated human platelets into LPS-primed SCID mice leads to accumulation of human platelets in the lung

**Figure 4:**
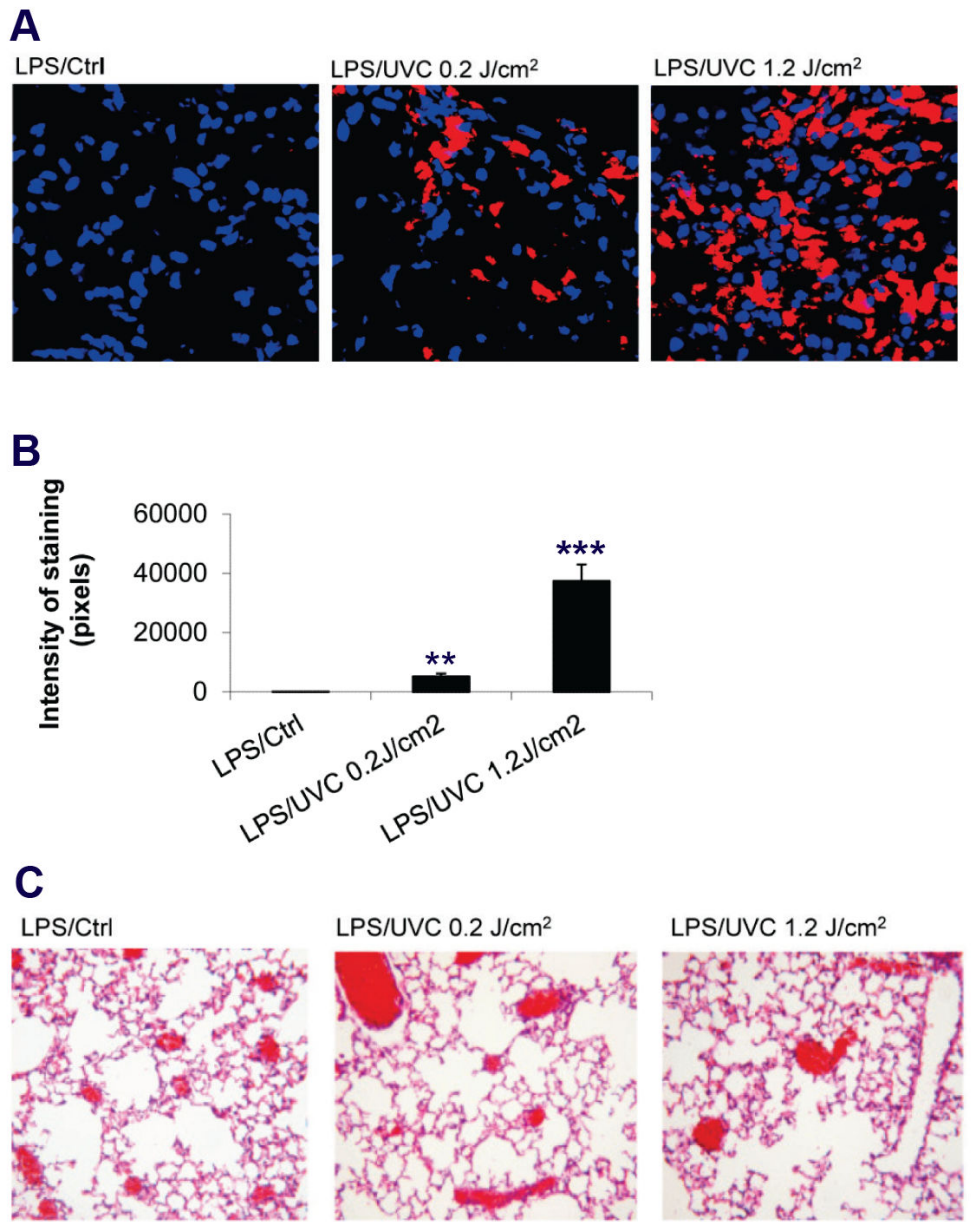
In vivo lung accumulation of UVC-irradiated HPs in the 2-event SCID mouse model. *A*) Mice were pretreated with an intraperitoneal injection of 3 mg/kg LPS 2 hours before intravenous infusion of untreated control or UVC-irradiated HPs at low (0.2 J/cm^2^) and high (1.2 J/cm^2^) doses, respectively. Shown are anti-human CD41 immunohistochemistry staining of lung frozen sections; *B*) Quantification of pixel intensity of anti-hCD41 staining of images shown in A. Mean ± SE, n=3; *C*) Lung histology from H&E staining of lung paraffin sections. Shown is a representative of three independent experiments.

Our previous work showed that UVB-irradiated human platelets accumulated in lungs of LPS-primed SCID mice [20]. Since UVC and UVB-platelets share similarities such as UV-induced platelet aggregation and PAC-1 binding, we explored the accumulation of UVC-platelets in lungs by using the specific anti-human CD41 monoclonal antibody. Our results show that UVC irradiation at a dose of 0.2 J/cm^2^ induced a mild increase in platelet staining in lung tissue as compared to control platelets, whereas increasing the UVC dose to 1.2 J/cm^2^ led to a marked increase in platelet lung accumulation, with detection of large platelet aggregates (Figure 4, A and B). However, in contrast to the local lung tissue response to the presence of UVB platelets [20], we did not observe a detectable increase in lung interstitial cellularity or edema with UVC platelet accumulation in the lung even when the platelets were exposed to a high dose of 1.2 J/cm^2^ UVC (Figure 4C). 

### UVC-irradiated platelets did not induce lung injury or chemokine response in LPS-primed SCID mice

**Figure 5:**
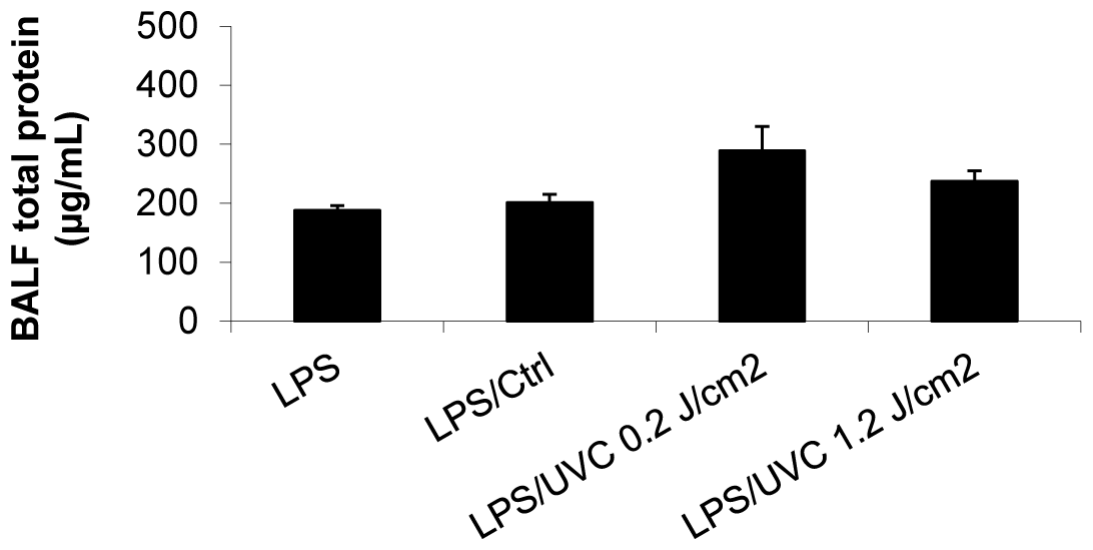
UVC-irradiated HPs did not induce lung injury in the 2-event SCID mouse model. Mice were treated as described above for the 2-event SCID mouse model. Bronchoalveolar fluid (BALF) was collected 1 hour after platelet infusion and the total protein concentration in BALF was measured using BCA protein assay. Mean ± SE, n=5-10.

**Figure 6:**
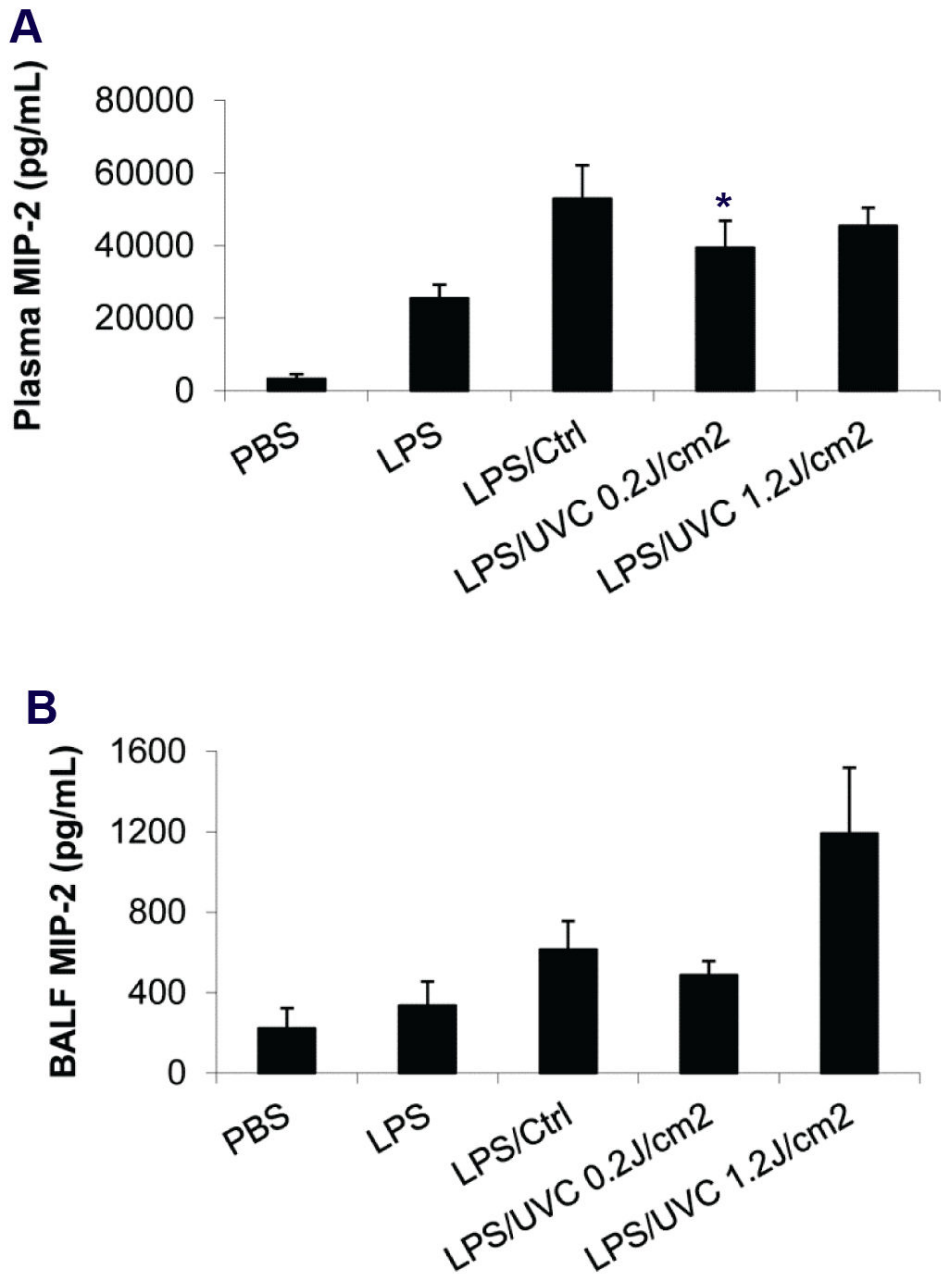
UVC-irradiated HPs failed to induce MIP-2 release in the 2-event SCID mouse model. 3 hours after HPs infusion mice were euthanized. Blood and BALF were collected and plasma was subsequently isolated as described in Materials and Methods. The concentrations of MIP-2 in plasma (*A*) and BALF (*B*) were measured using ELISA. Mean ± SE, n=4-5.

To examine whether transfusion of UVC-irradiated human platelets in LPS-primed SCID mice was associated with lung injury and release of macrophage inflammatory protein 2 (MIP-2) as we observed with UVB-irradiated platelets [20,21], we measured the total protein concentration in bronchoalveolar lavage fluid (BALF). An increase in protein accumulation in BALF is indicative of increased lung endothelial and alveolar cell permeability and has been referred to as the hallmark of acute lung injury [28]. Consistent with the minimal changes in lung histology in response to UVC-platelets (Figure 4C), no significant difference in BALF total protein level in LPS-primed mice transfused with UVC or control platelets was observed (Figure 5). Similarly, transfusion of UVC-platelets in LPS-primed mice did not induce further increase in plasma MIP-2 levels compared to mice transfused with control platelets (Figure 6A). Although BALF MIP-2 level showed a minor rise in a few mice transfused with high-dose UVC-irradiated platelets (1.2 J/cm^2^), due to the variability between animals the differences did not reach statistical significance when compared to mice transfused with control platelets (Figure 6B).

## Discussion

This study was designed to investigate the effect of UVC irradiation on platelet activation and aggregation and to explore whether changes in platelet in vitro quality correlated with in vivo recovery and pulmonary adverse events in LPS-pretreated recipient animals. 

The dose we chose for UVC was 0.2 J/cm^2^ based on recent modifications in the commercial UVC pathogen reduction system (THERAFLEX) [11,29] which is lower than the previously used commercial dose of 0.3 J/cm^2^ [12,29]. The reduced dose of 0.2 J/cm^2^ UVC has a reported slight impact on platelet activation and metabolic activity [11,29] while the pathogen reduction efficacy is claimed to be comparable [12,29]. 

In our experimental setup, 10 mL of platelets suspended in 65 % additive solution (PAS III) was added to a polypropylene container, resulting in a suspension depth of 1.2 mm. The intensity of the UVC light was controlled by adjusting the distance between the UVC lamp and the platelet suspension, so an exposure of 30 seconds and 3 minutes resulted in a UVC dose of 0.2 and 1.2 J/cm^2^, respectively. Samples were mixed thoroughly before exposing to UVC light. We assumed the small suspension depth and the short exposure time should allow efficient UVC light penetration in the platelet samples without shaking. Our system differs from the commercial application of UVC light in that the platelets are exposed to the light directly without it passing through the platelet bag plastic which may have an effect on the dose delivered and in our system is open to outside air as opposed to platelets being irradiated in a gas permeable bag. 

Verhaar R et al. previously reported a UVC dose of 0.15 J/cm^2^ reduced the platelet count immediately after irradiation [24]. Consistent with this finding, our results show that UVC exposure induced significant platelet microaggregation and doublet formation immediately after irradiation in a dose-dependent manner. At a UVC dose of 0.4 J/cm^2^, about 50 % decrease in single platelet count was observed, whereas reducing the dose to 0.2 J/cm^2^ resulted in a much milder (10 %) negative effect on the platelet count. While the spontaneous reduction in the platelet count at the low dose of 0.2 J/cm^2^ was minor, the potentiation of agonist-induced aggregation by this dose of UVC was quite pronounced. Even with low concentrations of agonist the UVC platelets reached near maximal aggregation. The potentiation of aggregation also correlated with a marked increase in UVC irradiation induced PAC-1 binding and was similar to the potentiation of ADP induced aggregation with UVB exposed platelets we reported previously [21]. Further increase in the UVC dose from 0.2 to 1.2 J/cm^2^ did not change the extent of PAC-1 binding on UVC-irradiated platelets, although it caused a more pronounced reduction in the single platelet count. The dose-dependent reduction in single platelet counts observed in our experiments is likely due to spontaneous formation of larger platelet aggregates, which were readily visible in the platelet suspension particularly at higher UVC doses.

In contrast to what we reported previously with UVB treatment of platelets [20], there was no detectable effect on platelet P-selectin expression when platelets were exposed to either 0.2 or 1.2 J/cm^2^ UVC irradiation. Verhaar R et al. reported that UVC induced platelet aggregation was the result of αIIbβ3 activation [24] which was independent of general platelet activation. The effects of UVC were shown to be due to a direct effect on αIIbβ3 through photolysis of disulfide bonds and caused a conformation change recognized by PAC-1 that lead to fibrinogen binding. P-selectin expression on platelet surface results from its translocation from intracellular granules to the external membrane when platelets become activated and secrete contents of their granules [30]. The lack of P-selectin expression after UVC treatment suggests that the platelets did not become activated and, as previously shown, the UVC-induced activation of αIIbβ3 is independent of intracellular signaling [24]. This is also consistent with the recent clinical trial results to evaluate the effect of UVC-treatment (0.3 J/cm^2^) on platelet in vitro function where P-selectin expression in treated platelets was not higher when compared to untreated control platelets [15]. It is of interest that a conformational change in αIIbβ3 did not lead to outside in signaling to produce platelet activation [31]. A conformational change sufficient to allow PAC-1 binding can lead to P-selectin expression in cases where integrin clustering and activation through the ADP receptor is present [32]. However, even high doses of UVC failed to increase P-selectin expression which suggests that the transmission of the conformation change through the transmembrane regions of αIIbβ3, which may be involved in the secondary signal generation [31], may also be different for integrin antagonists and for UVC irradiation. Indeed, we found pretreating platelets with clopidogrel, a P2Y12 ADP receptor inhibitor [33], prior to UVC irradiation caused about 10% decrease in the PAC-1 binding and ADP-induced aggregation in UVC platelets (Figure S1 and S2). This suggests that ADP mediated intraplatelet signaling does not play a major role in the activation of αIIbβ3 in UVC irradiated platelets.

Our study revealed an approximately 35 % lower in vivo recovery for 0.2 J/cm^2^ UVC-irradiated platelets than for control platelets at 2 and 4 hours after platelet transfusion in SCID mice. In comparison, UVB-irradiated platelets had 77.6 % and 94.5 % lower recovery at doses of 1.2 and 2.4 J/cm^2^ at these time points [20] indicating that UVC-irradiated platelets had milder reduction of in vivo recovery. Considering that UVC light at 0.2 J/cm^2^ induced similar levels of platelet integrin αIIbβ3 activation (PAC-1 binding) and potentiation of ADP-induced platelet aggregation as we previously reported with 2.4 J/cm^2^ UVB light [20,21], the difference in in vivo recovery was surprising. A major recognizable difference between UVC and UVB irradiated platelets appears to be the general activation of platelets by UVB as compared to selective activation of αIIbβ3 by UVC. The expression of P-selectin and/or other consequences of activation may contribute to the more rapid removal of UVB platelets from circulation [34].

UVB irradiated platelets accumulate in the lungs of LPS pretreated SCID mice and mediate acute lung injury (ALI) [20-22]. UVC irradiation at a dose of 1.2 J/cm^2^, caused similar levels of platelet lung accumulation as did UVB irradiation at a dose of 2.4 J/cm^2^ (Figure S3)[20,22]. Both treatments modify the αIIbβ3 conformation, lead to fibrinogen binding and potentiate platelet aggregation. However, UVC and UVB irradiated platelets differ strikingly in the consequences of their lung accumulation. The presence of UVB platelets in the lungs leads to an increase in the inflammatory cytokine MIP-2 levels in bronchoalveolar fluid (BALF) and plasma and to an increase in protein and leukocytes in the BALF signifying the generation of acute lung injury (ALI) [20-22]. In contrast, the UVC platelets accumulated in the lungs to a similar extent but did not lead to an increase in MIP-2 or to leakage of protein into the BALF in the same time frame as for UVB platelets even when the platelets were exposed to a high UVC dose of 1.2 J/cm^2^ Whether this is the consequence of UVB platelets being activated and expressing P-selectin or to timing of the response or to other aspects of activation is not clear, but it does indicate that platelet accumulation in the lung and subsequent lung damage are due to different and separate mechanisms. Identification of the mechanisms by which platelets accumulated in the lungs mediate lung damage will require further investigation. 

## Supporting Information

Figure S1 **Effect of clopidogrel on ADP induced platelet aggregation in UVC platelets.** HPs at 1 x 106/µL were pretreated with 100 µM clopidogrel (filled triangle) or DMSO(filled squaure) prior to exposing to 0.2 J/cm^2^ UVC illumination. In vitro aggregation assay was subsequently performed in the presence of increasing concentrations of ADP and compared to untreated control platelets (open circle). Y axis represents percent of maximal aggregation (%MA). Mean ± SE, n=3.
(TIF)

Figure S2 **Effect of clopidogrel on platelet activation markers in UVC platelets.** HPs at 1 x 106/µL were pretreated with 100 µM clopidogrel or DMSO prior to exposing to 0.2 or 1.2 J/cm^2^ UVC illumination. Cells were subsequently stained with a combination of CD41a-FITC and CD62P-PE (A) or with PAC1-FITC (B) and analyzed by flow cytometry. Shown is a representative of three independent experiments.
(TIF)

Figure S3 **In vivo lung accumulation of UVC- and UVB-irradiated HPs in the 2-event SCID mouse model.** A) Mice were pretreated with an intraperitoneal injection of 3 mg/kg LPS 2 hours before intravenous infusion of untreated HPs , or UVC-irradiated HPs at low (0.2 J/cm^2^) and high (1.2 J/cm^2^) doses, or UVB-irradiated HPs at 2.4 J/cm^2^, respectively. Shown are anti-human CD41 immunofluorescence staining of lung frozen sections; B) Quantification of pixel intensity of anti-hCD41 staining of images shown in A. Mean ± SE, n=3.
(TIF)

## References

[1] Hillyer CD, Josephson CD, Blajchman MA, Vostal JG, Epstein JS et al. (2003) Bacterial contamination of blood components: risks, strategies, and regulation: joint ASH and AABB educational session in transfusion medicine. Hematology Am Soc Hematol Educ Program: 575-589. 14633800 10.1182/asheducation-2003.1.575

[2] Montag T (2008) Strategies of bacteria screening in cellular blood components. Clin Chem Lab Med 46: 926-932. PubMed: 18624615. 18624615 10.1515/CCLM.2008.176

[3] Council of Europe (2002) Guide to the preparation, use and quality assurance of blood components, 8th ed. Strasbourg: Council of Europe Publishing.

[4] Lockwood WB, Leonard J, Liles S Chapter 9: Storage, Monitoring, Pretransfusion Processing, and Distribution of Blood Components. Technical Manual, 16th ed. AABB.

[5] Stramer SL, Hollinger FB, Katz LM, Kleinman S, Metzel PS et al. (2009) Emerging infectious disease agents and their potential threat to transfusion safety. Transfusion 49 (Suppl 2): 1S-29S. doi:10.1111/j.1537-2995.2009.02279.x. PubMed: 19686562. 19686562

[6] Irsch J, Lin L (2011) Pathogen Inactivation of Platelet and Plasma Blood Components for Transfusion Using the INTERCEPT Blood System. Transfus Med Hemother 38: 19-31. doi:10.1159/000323937. PubMed: 21779203. 21779203 PMC3132977

[7] Marschner S, Goodrich R (2011) Pathogen Reduction Technology Treatment of Platelets, Plasma and Whole Blood Using Riboflavin and UV Light. Transfus Med Hemother 38: 8-18. doi:10.1159/000324160. PubMed: 21779202. 21779202 PMC3132976

[8] Reddy HL, Dayan AD, Cavagnaro J, Gad S, Li J et al. (2008) Toxicity testing of a novel riboflavin-based technology for pathogen reduction and white blood cell inactivation. Transfus Med Rev 22: 133-153. doi:10.1016/j.tmrv.2007.12.003. PubMed: 18353253. 18353253

[9] Ciaravino V, McCullough T, Cimino G, Sullivan T (2003) Preclinical safety profile of plasma prepared using the INTERCEPT Blood System. Vox Sang 85: 171-182. doi:10.1046/j.1423-0410.2003.00351.x. PubMed: 14516447. 14516447

[10] Teitel JM (2000) Viral safety of haemophilia treatment products. Ann Med 32: 485-492. doi:10.3109/07853890009002024. PubMed: 11087169. 11087169

[11] Seltsam A, Müller TH (2011) UVC Irradiation for Pathogen Reduction of Platelet Concentrates and Plasma. Transfus Med Hemother 38: 43-54. doi:10.1159/000323845. PubMed: 21779205. 21779205 PMC3132979

[12] Mohr H, Steil L, Gravemann U, Thiele T, Hammer E et al. (2009) A novel approach to pathogen reduction in platelet concentrates using short-wave ultraviolet light. Transfusion 49: 2612-2624. doi:10.1111/j.1537-2995.2009.02334.x. PubMed: 19682340. 19682340

[13] Harm W (1980) Biology effects of ultraviolet radiation. Cambridge: Cambridge, UK.

[14] Picker SM, Schneider V, Oustianskaia L, Gathof BS (2009) Cell viability during platelet storage in correlation to cellular metabolism after different pathogen reduction technologies. Transfusion 49: 2311-2318. doi:10.1111/j.1537-2995.2009.02316.x. PubMed: 19624608. 19624608

[15] Bashir S, Cookson P, Wiltshire M, Hawkins L, Sonoda L et al. (2012) Pathogen inactivation of platelets using ultraviolet C light: effect on in vitro function and recovery and survival of platelets. Transfusion 53: 990–1000. PubMed: 22905813. 22905813 10.1111/j.1537-2995.2012.03854.x

[16] Snyder E, Raife T, Lin L, Cimino G, Metzel P et al. (2004) Recovery and life span of 111indium-radiolabeled platelets treated with pathogen inactivation with amotosalen HCl (S-59) and ultraviolet A light. Transfusion 44: 1732-1740. doi:10.1111/j.0041-1132.2004.04145.x. PubMed: 15584988. 15584988

[17] AuBuchon JP, Herschel L, Roger J, Taylor H, Whitley P et al. (2005) Efficacy of apheresis platelets treated with riboflavin and ultraviolet light for pathogen reduction. Transfusion 45: 1335-1341. doi:10.1111/j.1537-2995.2005.00202.x. PubMed: 16078923. 16078923

[18] Snyder E, McCullough J, Slichter SJ, Strauss RG, Lopez-Plaza I et al. (2005) Clinical safety of platelets photochemically treated with amotosalen HCl and ultraviolet A light for pathogen inactivation: the SPRINT trial. Transfusion 45: 1864-1875. doi:10.1111/j.1537-2995.2005.00639.x. PubMed: 16371039. 16371039

[19] Cazenave JP, Follea G, Bardiaux L, Boiron JM, Lafeuillade B et al. (2010) A randomized controlled clinical trial evaluating the performance and safety of platelets treated with MIRASOL pathogen reduction technology. Transfusion 50: 2362-2375. doi:10.1111/j.1537-2995.2010.02694.x. PubMed: 20492615. 20492615

[20] Gelderman MP, Chi X, Zhi L, Vostal JG (2011) Ultraviolet B light-exposed human platelets mediate acute lung injury in a two-event mouse model of transfusion. Transfusion 51: 2343-2357. doi:10.1111/j.1537-2995.2011.03135.x. PubMed: 21492179. 21492179

[21] Zhi L, Chi X, Gelderman MP, Vostal JG (2012) Activation of platelet protein kinase C by ultraviolet light B mediates platelet transfusion-related acute lung injury in a two-event animal model. Transfusion. 10.1111/j.1537-2995.2012.03811.x 22853798

[22] Chi X, Zhi L, Gelderman MP, Vostal JG (2012) Host platelets and, in part, neutrophils mediate lung accumulation of transfused UVB-irradiated human platelets in a mouse model of acute lung injury. PLOS ONE 7: e44829. doi:10.1371/journal.pone.0044829. PubMed: 23028636. 23028636 PMC3446987

[23] Kirkeby S, Thomsen CE (2005) Quantitative immunohistochemistry of fluorescence labelled probes using low-cost software. J Immunol Methods 301: 102-113. doi:10.1016/j.jim.2005.04.006. PubMed: 15982663. 15982663

[24] Verhaar R, Dekkers DW, De Cuyper IM, Ginsberg MH, de Korte D et al. (2008) UV-C irradiation disrupts platelet surface disulfide bonds and activates the platelet integrin alphaIIbbeta3. Blood 112: 4935-4939. doi:10.1182/blood-2008-04-151043. PubMed: 18796633. 18796633 PMC2597600

[25] Shattil SJ, Cunningham M, Hoxie JA (1987) Detection of activated plaelets in whole blood using activation-dependent monoclonal antibodies and flow cytometry. Blood 70: 307-315. PubMed: 3297204. 3297204

[26] Shattil SJ, Hoxie JA, Cunningham M, Brass LF (1985) Changes in te platelet membrane glycoprotin IIb-IIIa complex during platelet activation. J Biol Chem 260: 11107-11114. PubMed: 2411729. 2411729

[27] Taub R, Gould RJ, Garsky VM, Ciccarone TM, Hoxie J et al. (1989) A monoclonal antibody against the platelet fibrinogen receptor contains a sequence that mimics a receptor recognition domain in fibrinogen. J Biol Chem 264: 259-265. PubMed: 2909518. 2909518

[28] Toy P, Popovsky MA, Abraham E, Ambruso DR, Holness LG et al. (2005) Transfusion-related acute lung injury: definition and review. Crit Care Med 33: 721-726. doi:10.1097/01.CCM.0000159849.94750.51. PubMed: 15818095. 15818095

[29] Sandgren P, Tolksdorf F, Struff WG, Gulliksson H (2011) In vitro effects on platelets irradiated with short-wave ultraviolet light without any additional photoactive reagent using the THERAFLEX UV-Platelets method. Vox Sang 101: 35-43. doi:10.1111/j.1423-0410.2010.01454.x. PubMed: 21175668. 21175668

[30] Lasky LA (1992) Selectins: interpreters of cell-specific carbohydrate information during inflammation. Science 258: 964-969. doi:10.1126/science.1439808. PubMed: 1439808. 1439808

[31] Shattil SJ, Kim C, Ginsberg MH (2010) The final steps of integrin activation: the end game. Nat Rev Mol Cell Biol 11: 288-300. doi:10.1038/nrm2871. PubMed: 20308986. 20308986 PMC3929966

[32] Bassler N, Loeffler C, Mangin P, Yuan Y, Schwarz M et al. (2007) A mechanistic model for paradoxical platelet activation by ligand-mimetic alphaIIb beta3 (GPIIb/IIIa) antagonists. Arterioscler Thromb Vasc Biol 27: e9-15. doi:10.1161/01.ATV.0000255307.65939.59. PubMed: 17170374. 17170374

[33] Bertrand ME, Rupprecht HJ, Urban P, Gershlick AH (2000) Double-blind study of the safety of clopidogrel with and without a loading dose in combination with aspirin compared with ticlopidine in combination with aspirin after coronary stenting: the clopidogrel aspirin stent international cooperative study (CLASSICS). Circulation 102: 624-629. doi:10.1161/01.CIR.102.6.624. PubMed: 10931801. 10931801

[34] Merten M, Thiagarajan P (2000) P-selectin expression on platelets determines size and stability of platelet aggregates. Circulation 102: 1931-1936. doi:10.1161/01.CIR.102.16.1931. PubMed: 11034941. 11034941

