# Supplementary figures and images for: In Vitro and In Vivo Characterization of Ultraviolet Light C-Irradiated Human Platelets in a 2 Event Mouse Model of Transfusion
Source: PLoS One. 2013 Nov 1;8(11):e79869. doi: 10.1371/journal.pone.0079869 (PMC3815158; doi:10.1371/journal.pone.0079869)

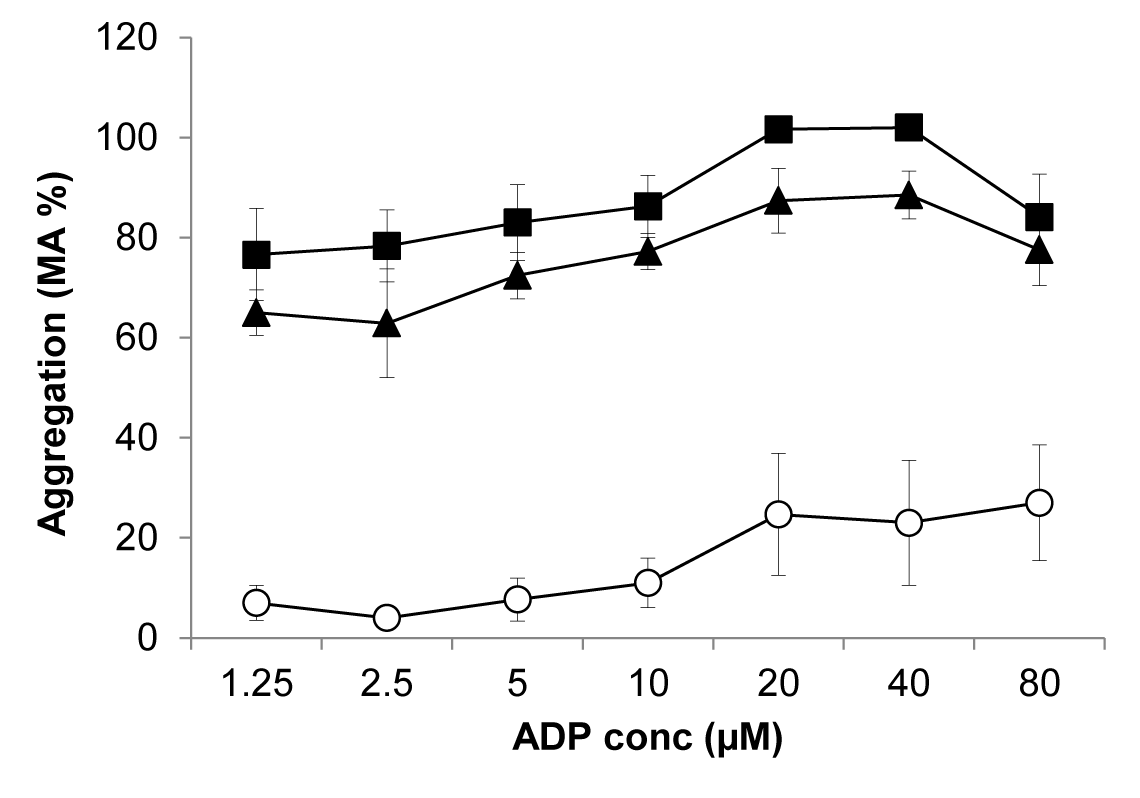

Supplement: Figure S1 — Effect of clopidogrel on ADP induced platelet aggregation in UVC platelets. HPs at 1 x 10^6^/µL were pretreated with 100 µM clopidogrel (filled triangle) or DMSO(filled squaure) prior to exposing to 0.2 J/cm2 UVC illumination. In vitro aggregation assay was subsequently performed in the presence of increasing concentrations of ADP and compared to untreated control platelets (open circle). Y axis represents percent of maximal aggregation (%MA). Mean ± SE, n=3. (TIF) [file pone.0079869.s001.tif]

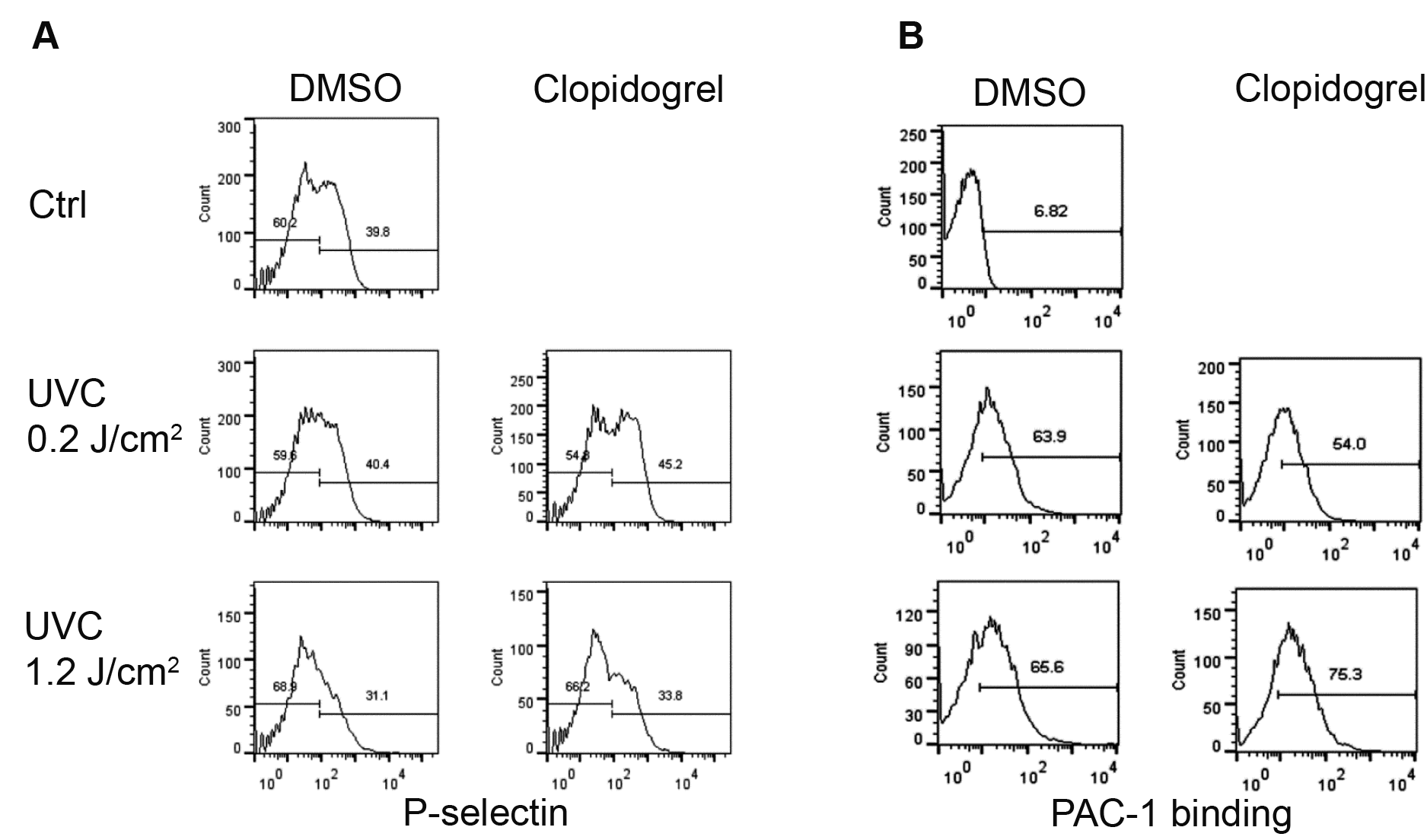

Supplement: Figure S2 — Effect of clopidogrel on platelet activation markers in UVC platelets. HPs at 1 x 10^6^/µL were pretreated with 100 µM clopidogrel or DMSO prior to exposing to 0.2 or 1.2 J/cm2 UVC illumination. Cells were subsequently stained with a combination of CD41a-FITC and CD62P-PE (A) or with PAC1-FITC (B) and analyzed by flow cytometry. Shown is a representative of three independent experiments. (TIF) [file pone.0079869.s002.tif]

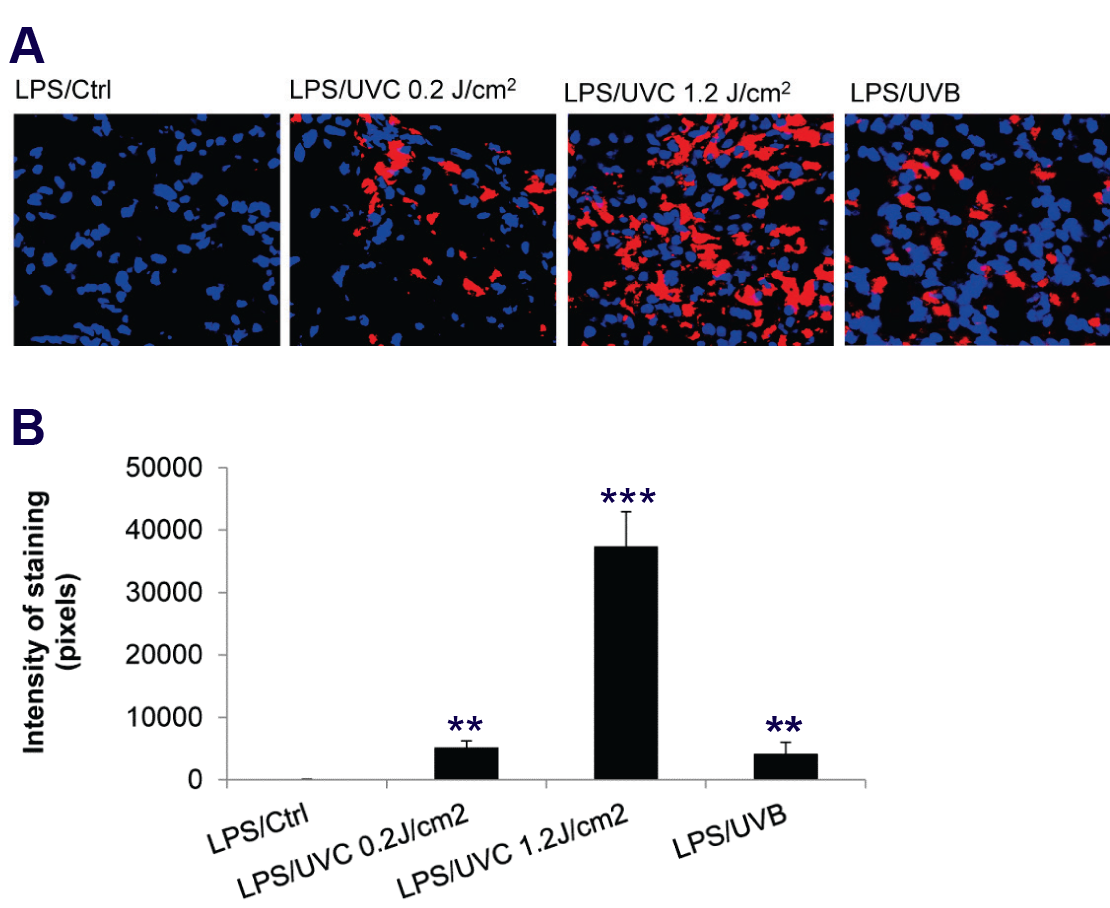

Supplement: Figure S3 — In vivo lung accumulation of UVC- and UVB-irradiated HPs in the 2-event SCID mouse model. A) Mice were pretreated with an intraperitoneal injection of 3 mg/kg LPS 2 hours before intravenous infusion of untreated HPs , or UVC-irradiated HPs at low (0.2 J/cm2) and high (1.2 J/cm2) doses, or UVB-irradiated HPs at 2.4 J/cm2, respectively. Shown are anti-human CD41 immunofluorescence staining of lung frozen sections; B) Quantification of pixel intensity of anti-hCD41 staining of images shown in A. Mean ± SE, n=3. (TIF) [file pone.0079869.s003.tif]
